# Dynamic Control of Mitochondrial Ca^2+^ Levels as a Survival Strategy of Cancer Cells

**DOI:** 10.3389/fcell.2021.614668

**Published:** 2021-02-04

**Authors:** Corina T. Madreiter-Sokolowski, Benjamin Gottschalk, Armin A. Sokolowski, Roland Malli, Wolfgang F. Graier

**Affiliations:** ^1^Molecular Biology and Biochemistry, Gottfried Schatz Research Center, Medical University of Graz, Graz, Austria; ^2^Department of Health Sciences and Technology, ETH Zurich, Schwerzenbach, Switzerland; ^3^Department of Dental Medicine and Oral Health, Medical University of Graz, Graz, Austria; ^4^BioTechMed Graz, Graz, Austria

**Keywords:** cancer cells, mitochondrial Ca^2+^ homeostasis, mitochondrial-ER interaction, uncoupling protein 2, ER stress

## Abstract

Cancer cells have increased energy requirements due to their enhanced proliferation activity. This energy demand is, among others, met by mitochondrial ATP production. Since the second messenger Ca^2+^ maintains the activity of Krebs cycle dehydrogenases that fuel mitochondrial respiration, proper mitochondrial Ca^2+^ uptake is crucial for a cancer cell survival. However, a mitochondrial Ca^2+^ overload induces mitochondrial dysfunction and, ultimately, apoptotic cell death. Because of the vital importance of balancing mitochondrial Ca^2+^ levels, a highly sophisticated machinery of multiple proteins manages mitochondrial Ca^2+^ homeostasis. Notably, mitochondria sequester Ca^2+^ preferentially at the interaction sites between mitochondria and the endoplasmic reticulum (ER), the largest internal Ca^2+^ store, thus, pointing to mitochondrial-associated membranes (MAMs) as crucial hubs between cancer prosperity and cell death. To investigate potential regulatory mechanisms of the mitochondrial Ca^2+^ uptake routes in cancer cells, we modulated mitochondria–ER tethering and the expression of UCP2 and analyzed mitochondrial Ca^2+^ homeostasis under the various conditions. Hence, the expression of contributors to mitochondrial Ca^2+^ regulation machinery was quantified by qRT-PCR. We further used data from The Cancer Genome Atlas (TCGA) to correlate these *in vitro* findings with expression patterns in human breast invasive cancer and human prostate adenocarcinoma. ER-mitochondrial linkage was found to support a mitochondrial Ca^2+^ uptake route dependent on uncoupling protein 2 (UCP2) in cancer cells. Notably, combined overexpression of Rab32, a protein kinase A-anchoring protein fostering the ER-mitochondrial tethering, and UCP2 caused a significant drop in cancer cells' viability. Artificially enhanced ER-mitochondrial tethering further initiated a sudden decline in the expression of UCP2, probably as an adaptive response to avoid mitochondrial Ca^2+^ overload. Besides, TCGA analysis revealed an inverse expression correlation between proteins stabilizing mitochondrial-ER linkage and UCP2 in tissues of human breast invasive cancer and prostate adenocarcinoma. Based on these results, we assume that cancer cells successfully manage mitochondrial Ca^2+^ uptake to stimulate Ca^2+^-dependent mitochondrial metabolism while avoiding Ca^2+^-triggered cell death by fine-tuning ER-mitochondrial tethering and the expression of UCP2 in an inversed manner. Disruption of this equilibrium yields cancer cell death and may serve as a treatment strategy to specifically kill cancer cells.

## Introduction

The Ca^2+^ ion is a potent and versatile cellular messenger to regulate mitochondrial functions. In mitochondria, Ca^2+^ elevations boost the activity of the mitochondrial electron transport chain (ETC) by stimulating Ca^2+^-dependent dehydrogenases of the Krebs cycle (Denton et al., 1975), modulates mitochondrial membrane potential, and induces apoptotic cell death upon mitochondrial Ca^2+^ overload (Madreiter-Sokolowski et al., 2017a). Accordingly, to balance mitochondrial Ca^2+^ homeostasis is of utmost importance for all cells. Contact sites between the ER and mitochondria are established by so-called mitochondria associated ER membranes (MAMs) that ensure locally restricted and highly controlled ion transfer between these organelles (Simmen and Herrera-Cruz, 2018). Within this region, a sophisticated toolkit of proteins executes and controls the sequestration, transfer, and extrusion of Ca^2+^ ions across the two mitochondrial membranes. In the outer mitochondrial membrane (OMM), the voltage-dependent anion channel (VDAC) is activated by elevated cytosolic Ca^2+^ levels within MAM regions, allowing a great permeability to Ca^2+^ (Bathori et al., 2006). In contrast, Ca^2+^ transport across the inner mitochondrial membrane (IMM) is highly restricted and sophisticated regulated. Thereby, Ca^2+^ concentrations exceeding 10 μM in regions with MAMs (Giacomello et al., 2010; Patergnani et al., 2011) relieve the gatekeeper proteins mitochondrial Ca^2+^ uptake 1 and 2 (MICU1 and MICU2) and allow Ca^2+^ flux through the pore-forming couple of mitochondrial Ca^2+^ uniporter (MCU) (Madreiter-Sokolowski et al., 2019a) and the essential MCU regulator (EMRE) (Sancak et al., 2013). The mitochondrial uniporter complex (MCUC) further consists of the dominant-negative pore-forming subunit MCUb (Raffaello et al., 2013) and the scaffold factor MCU regulator 1 (MCUR1) (Tomar et al., 2016). However, mitochondria can sequester Ca^2+^ even in the absence of MCU, thus, indicating alternative pathways that directly or indirectly affect mitochondrial Ca^2+^ uptake (Bisbach et al., 2020). The Ca^2+^/H^+^ exchanger leucine-zipper and EF-hand containing transmembrane protein 1 (LETM1) modulates mitochondrial Ca^2+^ homeostasis (Jiang et al., 2009), metabolic signaling (Doonan et al., 2014) and cristae organization (Nakamura et al., 2020), while it does not mediate mitochondrial Ca^2+^ extrusion (De Marchi U. et al., 2014). Another contributor to mitochondrial Ca^2+^ uptake is the uncoupling protein 2 (UCP2) (Trenker et al., 2007). Under conditions of elevated protein methyltransferase 1 (PRMT1), as the case in cancer or aging (Blanc and Richard, 2017), UCP2 interacts with PRMT1-methylated MICU1 and re-establishes its Ca^2+^ sensitivity resulting in a normalization of mitochondrial Ca^2+^ uptake (Madreiter-Sokolowski et al., 2016a). Besides regulating the sequestration of Ca^2+^ into the mitochondria, mitochondrial Ca^2+^ extrusion via the mitochondrial Na^+^/Ca^2+^ exchanger (NCLX) is of utmost importance (Palty et al., 2010; Sekler, 2015).

In cancer cells, mitochondrial Ca^2+^ homeostasis appears to be tightly balanced between boosting mitochondrial ATP production to meet enhanced energy demand due to high proliferation activity and running the risk of mitochondrial Ca^2+^ overload-induced cell death. Constitutive Ca^2+^ flux from the ER to mitochondria was reported to be crucial for cancer cells (Cardenas et al., 2016). Moreover, close mitochondrial-ER interplay was shown to boost energy metabolism, favoring cancer cells' survival (Madreiter-Sokolowski et al., 2016b). However, extensive tethering between mitochondria and ER structures introduces the risk for mitochondrial Ca^2+^ overload, potentially triggered by compounds boosting mitochondrial Ca^2+^ uptake, such as resveratrol (Madreiter-Sokolowski et al., 2016b). Under conditions of mitochondrial Ca^2+^ overload, the opening of the mitochondrial permeability transition pore (mPTP) allows uncontrolled release of Ca^2+^ and apoptotic factors like cytochrome C into the cytoplasm (De Marchi E. et al., 2014). Recent findings suggest that it is dependent on the cancer type and cancer stage whether a proper ER-mitochondrial Ca^2+^ transfer can either boost pro-tumorigenic mechanisms or exert anti-tumorigenic effects by restoring apoptosis sensitivity. Notably, oncogenes and tumor suppressors located at the interaction sites between ER and mitochondria were shown to modulate the ER-mitochondrial Ca^2+^ flux (Kerkhofs et al., 2018). ER Ca^2+^ release via the 1,4,5-trisphosphate receptor type 2 (IP3R2) was also shown to trigger MCU-dependent mitochondrial Ca^2+^ accumulation during oncogene-induced senescence (Wiel et al., 2014). IP3R-mediated release of Ca^2+^ from the ER, followed by MCU-mediated mitochondrial Ca^2+^ uptake, was also associated with the induction of paraptosis, a cell death mode linked to extensive vacuolization, triggered by the tripterine celastrol (Yoon et al., 2014). Besides, cancer cells tune their mitochondrial Ca^2+^ uptake very tightly by alterations in the expression and function of proteins involved in mitochondrial Ca^2+^ uptake and extrusion, including MCU, MICU1, MICU2, MCUR1, and NCLX (Delierneux et al., 2020). Notably, global genomic analysis identified loss of heterozygosity for MCU and MICU1 in human pancreatic cancer tissues, hinting to an involvement of these genes in cancer progression (Long et al., 2015). The expression of MCU was found to be upregulated in several cancer types including breast cancer and liver cancer. Knockdown of MCU in respective cancer cells resulted in decreased migration an invasion, potentiation of compound-induced cell death and metabolic restructuring (Delierneux et al., 2020). A study using triple-negative breast cancer xenografts demonstrated that downregulation of MCU hampers cell motility, invasiveness, and tumor progression and revealed a positive correlation between MCU expression and hypoxia-inducible factor-1α (HIF-1α), hinting to an MCU-associated regulation of cancer progression via HIF-1α (Tosatto et al., 2016). MCU was also shown to be crucial for migration of MDA-MB-231 cells by enabling a functional store-operated Ca^2+^ entry (SOCE) (Tang et al., 2015). While silencing of MCU did not affect proliferation or cell viability of MDA-MB-231 cells (Curry et al., 2013; Hall et al., 2014), the ionophore ionomycin was found to induce caspase-independent cell death in MDA-MB-231 depleted of MCU (Curry et al., 2013). In addition, MCU-induced Ca^2+^ uptake was found to promote mitochondrial biogenesis and colorectal cancer growth (Liu et al., 2020). The crucial role of MCU regulators in cancer progression gets also obvious by the impact of MICU1 impairment, which results in the opening of MCU, enhanced mitochondrial Ca^2+^ uptake and ROS production in HeLa, and potentially boosts tumor growth (Marchi et al., 2019). Moreover, a study based on a xenograft tumor model revealed that targeting the enhancer of zeste homolog 2 (EZH2), known as a negative prognostic factor in most human cancers, regulates growth, and apoptosis of head and neck squamous cell carcinoma via MICU1, required to maintain stability of mitochondrial membrane potential (Zhou et al., 2015). Besides, mitochondrial Ca^2+^ uptake controlled by UCP2 and PRMT1 was found to boost devastating tumor growth by ensuring proper ATP biosynthesis while avoiding the risk of Ca^2+^ overload-induced cell death (Madreiter-Sokolowski et al., 2017b).

Here we report that the upregulation of UCP2 significantly reduces cancer cells' viability in case of enhanced ER-mitochondrial tethering. Moreover, overexpression of Rab32, a protein kinase A-anchoring protein fostering the ER-mitochondrial tethering (Bui et al., 2010), leads to a decline in the expression of UCP2, which points to an adaption strategy of cancer cells to escape mitochondrial Ca^2+^ overload-induced cell death. These *in vitro* findings were further supported by the inverse expression pattern between proteins stabilizing mitochondrial-ER linkage and UCP2 in human invasive breast cancer and pancreatic adenocarcinoma tissues.

Based on our present results, we assume that the tightly controlled mitochondrial Ca^2+^ homeostasis within mitochondrial-ER interaction sites is a potential target to kill cancer cells.

## Results

### Impact of UCP2 Dependent on Stable Mitochondrial-ER Interaction

**Figure 1:**
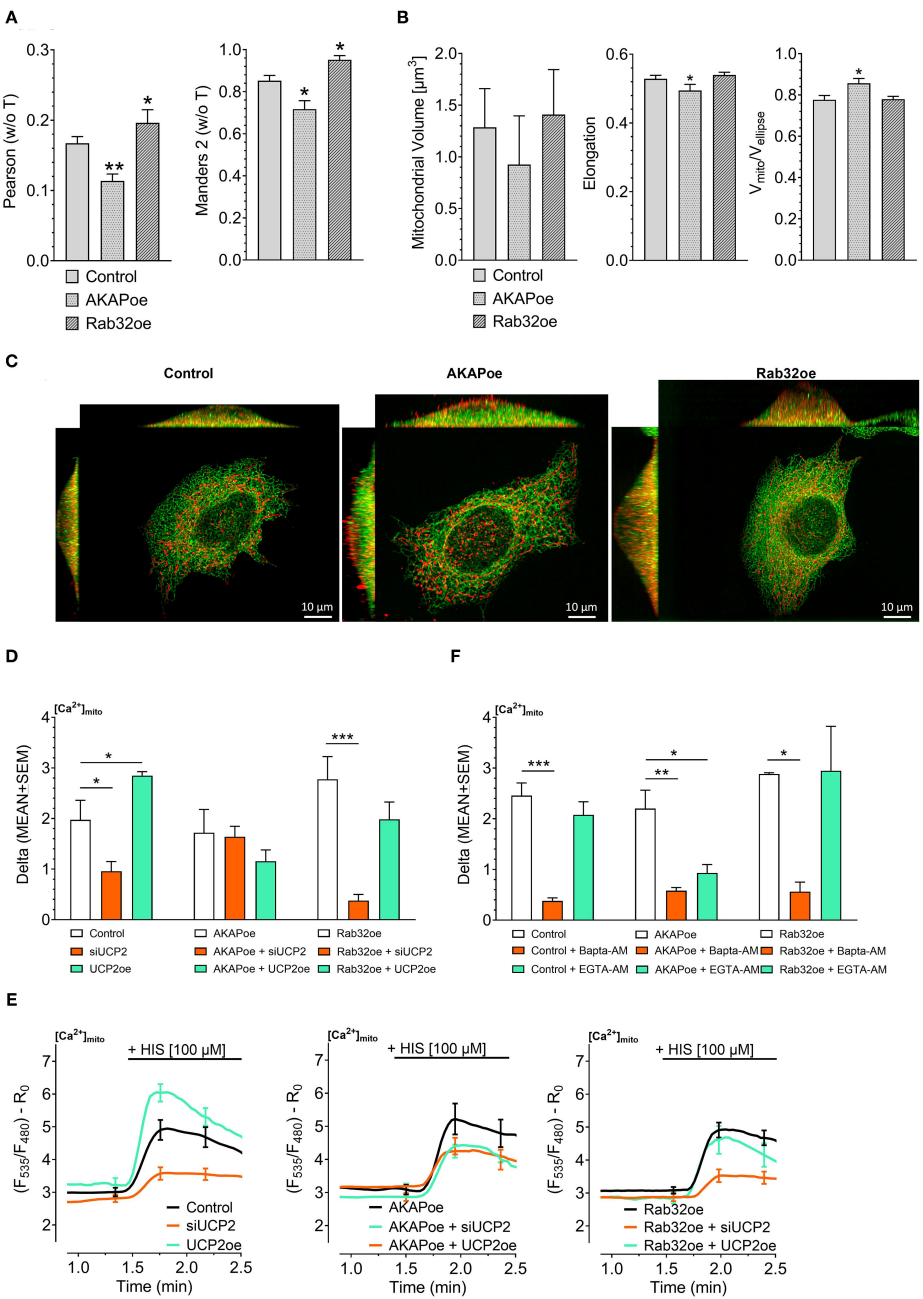
Impact of UCP2 on mitochondrial Ca^2+^ uptake under conditions of reduced or enhanced mitochondrial-ER interplay. *Left* bar graph shows Pearson coefficient analyzing colocalization of mitochondria and ER in control HeLa cells (*n* = 68/6), AKAP-RFP-CAAX expressing HeLa cells (*n* = 51/14), and Rab32 overexpressing HeLa cells (*n* = 68/4). *Right* bar graph reflects Manders 2 coefficient analyzing colocalization of mitochondria and ER in control HeLa cells (*n* = 68/6), AKAP-RFP-CAAX expressing HeLa cells (*n* = 51/14), and Rab32 overexpressing HeLa cells (*n* = 68/4) **(A)**. *Left* bar graph shows mitochondrial volume [μm^3^], *middle* bar graph elongation and, *right* bar graph V_mito_/V_ellipse_ of control HeLa cells (*n* = 68/6), AKAP-RFP-CAAX expressing HeLa cells (*n* = 51/14), and Rab32 overexpressing HeLa cells (*n* = 68/4) **(B)**. Representative x/y, x/z, and y/z maximum intensity projections of confocal z-stacks of control HeLa cells (*left*), AKAP-RFP-CAAX overexpressing HeLa (*middle*) or Rab32 overexpressing HeLa (*right*), expressing D1ER (green), and stained with MitoTrackerRed® CMXRos (red) **(C)**. Bar graphs represent mitochondrial Ca^2+^ uptake in response to 100 μM histamine in HeLa cells treated with control siRNA (*n* = 30/9), with siRNA against UCP2 (*n* = 26/7) or overexpressing UCP2 (*n* = 14/9) as well as in AKAP-RFP-CAAX overexpressing HeLa cells treated with control siRNA (*n* = 21/7), with siRNA against UCP2 (*n* = 20/7) or overexpressing UCP2 (*n* = 17/6) as well as in Rab32 overexpressing HeLa cells treated with control siRNA (*n* = 21/7), with siRNA against UCP2 (*n* = 21/7) or overexpressing UCP2 (*n* = 13/5) **(D)**. *Left* figure shows representative Ca^2+^ traces, obtained by using the organelle-targeted Ca^2+^ sensor 4mtD3cpv, of control HeLa cells (black curves), HeLa cells depleted of UCP2 (orange curves), and HeLa cells overexpressing UCP2 (green curves). *Middle* figure shows representative Ca^2+^ traces of AKAP-RFP-CAAX overexpressing HeLa (black curves), HeLa cells depleted of UCP2 (orange curves), and HeLa cells overexpressing UCP2 (green curves). *Right* figure shows representative Ca^2+^ traces of AKAP-RFP-CAAX overexpressing HeLa (black curves), HeLa cells depleted of UCP2 (orange curves), and HeLa cells overexpressing UCP2 (green curves) **(E)**. Bar graphs represent mitochondrial Ca^2+^ uptake in response to 100 μM histamine in control HeLa cells, untreated (*n* = 45/13), pretreated for 45 min with 2 μM of BAPTA-AM (*n* = 32/5) or with 2 μM of EGTA-AM (*n* = 33/5), in AKAP-RFP-CAAX overexpression HeLa cells, untreated (*n* = 56/20), pretreated for 45 min with 2 μM of BAPTA-AM (*n* = 40/9) or with 2 μM of EGTA-AM (*n* = 38/9), or in Rab32 overexpressing HeLa cells, untreated (*n* = 9/3), pretreated for 45 min with 2 μM of BAPTA-AM (*n* = 18/4) or with 2 μM of EGTA-AM (*n* = 12/4) **(F)**. Significant differences were assessed via one-way ANOVA and presented as specific *p*-values (*p ≤ 0.05, **p ≤ 0.01, ***p ≤ 0.001).

Previous work revealed that the source of Ca^2+^ that approaches the mitochondrial surface, either intracellular Ca^2+^ release or Ca^2+^ entering via plasma membrane Ca^2+^ channels, defines the type of mitochondrial Ca^2+^ uptake route (Waldeck-Weiermair et al., 2011). To simulate conditions of low mitochondrial-ER interaction, we overexpressed the AKAP-RFP-CAAX construct tagging mitochondria to the plasma membrane (Csordas et al., 2006; Naghdi et al., 2010). In contrast to such disruption of the mitochondria–ER contacts, overexpression of the protein kinase A-anchoring protein Rab32 was used to artificially enhance tethering between mitochondria and ER (Bui et al., 2010). Respective colocalization analysis confirmed reduced contact sites between mitochondria and ER in cells overexpressing AKAP-RFP-CAAX and increased mitochondrial-ER tethering upon Rab32 overexpression (Figures 1A,C). Mitochondrial morphology remained unchanged by Rab32 overexpression, while overexpression of AKAP-RFP-CAAX caused less elongated and branched mitochondria (Figure 1B). In experiments measuring mitochondrial matrix Ca^2+^ levels, knockdown of UCP2 decreased and overexpression of UCP2 increased mitochondrial Ca^2+^ uptake in response to histamine-induced ER Ca^2+^ depletion in control HeLa cells (Figures 1D,E). Manipulation of UCP2's expression level did not affect mitochondrial Ca^2+^ uptake in HeLa cells overexpressing AKAP-RFP-CAAX (AKAPoe). In contrast, depletion of UCP2 strongly diminished mitochondrial Ca^2+^ uptake in HeLa cells overexpressing Rab32. However, overexpression of UCP2 failed to boost mitochondrial Ca^2+^ uptake under this condition (Figures 1D,E). To demonstrate the functional impact of ER-Ca^2+^ crosstalk modulation, we performed mitochondrial Ca^2+^ measurements using different intracellular Ca^2+^ chelators, bis-aminophenoxy-tetraacetic acid (BAPTA-AM) and ethylene glycol-bis-tetraacetic acid (EGTA-AM). Since BAPTA-AM buffers Ca^2+^ much faster than EGTA-AM, the usage of these chelators allowed us to indirectly estimate the distance Ca^2+^ has to overcome when released from the ER and taken up by mitochondria. Under control conditions as well as in case of Rab32 overexpression, BAPTA-AM was able to significantly diminish mitochondrial Ca^2+^ uptake by efficient buffering of Ca^2+^ in the cytosol, while the slower acting EGTA-AM did not affect mitochondrial Ca^2+^ uptake significantly (Figure 1F). As shown in Figure 1A, AKAP-CAAX-RFP diminished the interaction between ER and mitochondria. BAPTA-AM as well as slower buffering EGTA-AM reduced mitochondrial Ca^2+^ uptake in HeLa overexpression AKAP-CAAX-RFP, pointing to an enlarged gap between ER and mitochondria (Figure 1F).

### UCP2 Expression and Mitochondrial-ER Communication as Crucial Determinants in Cancer Cell's Survival

**Figure 2:**
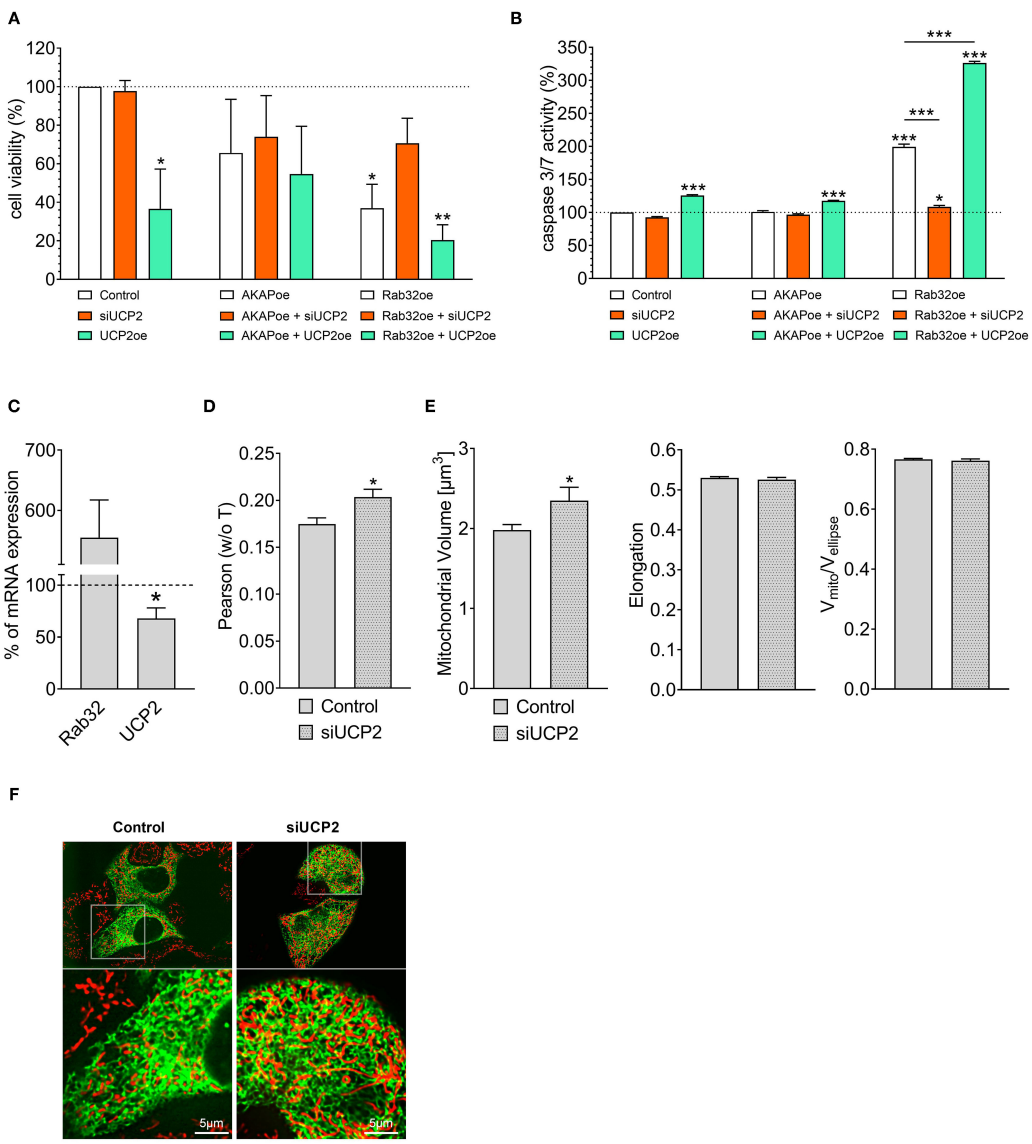
Interrelation between UCP2 expression and mitochondrial-ER communication. Cell viability of control HeLa cells, AKAP-RFP-CAAX expressing HeLa cells or Rab32 overexpressing HeLa cells with siRNA against UCP2 or UCP2 overexpressing, measured via Celltiter-Blue assay, and calculated as the percentage of viable cells normalized to control conditions (*n* = 3) **(A)**. Apoptotic caspase 3/7 activity of control HeLa cells, AKAP-RFP-CAAX expressing HeLa cells or Rab32 overexpressing HeLa cells with siRNA against UCP2 or UCP2 overexpressing, measured by Caspase 3/7-Glo assay and calculated as the percentage of viable cells normalized to control conditions (*n* = 3) **(B)**. mRNA expression ratios of Rab32 and UCP2 in HeLa cells overexpressing Rab32 in comparison to control HeLa cells (*n* = 3) **(C)**. Pearson coefficient showing colocalization of mitochondria and ER in siRNA treated control HeLa cells (*n* = 235/24) and HeLa cells with siRNA-induced knockdown of UCP2 (*n* = 144/17) **(D)**. *Left* bar graph reflects mitochondrial volume [μm^3^], *middle* bar graph elongation and *right* bar graph V_mito_/V_ellipse_ of siRNA treated control HeLa cells (*n* = 235/24) and HeLa cells with siRNA-induced knockdown of UCP2 (*n* = 144/17) **(E)**. Representative confocal images of siRNA treated control HeLa cells and HeLa cells with siRNA-induced knockdown of UCP2 expressing D1ER (green) and stained with MitoTrackerRed® CMXRos (red) **(F)**. Significant differences were assessed via one-way ANOVA or via unpaired *t-*test, if applicable, and presented as specific *p*-values (*p ≤ 0.05, **p ≤ 0.01, ***p ≤ 0.001).

Cell viability analysis confirmed our experience from live-cell imaging: Overexpression of UCP2 alone reduced cell viability (Figure 2A) and increased apoptotic caspase 3/7 activity in control HeLa cells (Figure 2B). Hence, UCP2's overexpression did not significantly affect cell viability (Figure 2A) in HeLa cells with reduced mitochondrial-ER tethering due to AKAP-RFP-CAAX expression. In contrast, overexpression of Rab32 significantly reduced cell viability (Figure 2A) and enhanced caspase 3/7 activity (Figure 2B) already without any manipulation of UCP2's expression level. Under conditions of enhanced ER–mitochondrial stability, overexpression of UCP2 even further reduced cell viability and significantly enhanced caspase 3/7 activity. In contrast, knockdown of UCP2 rescued cell viability and normalized caspase 3/7 activity in cells with increased mitochondria–ER tethering (Figures 2A,B). Notably, analysis of HeLa cells that survived Rab32 overexpression revealed a significant downregulation of UCP2 (Figure 2C). In contrast, siRNA-induced knockdown of UCP2 caused enhanced ER-mitochondrial colocalization in HeLa cells (Figures 2D,F), mitochondrial volume got increased, while the other parameters of mitochondrial morphology remained largely unchanged (Figure 2E). These data demonstrate the susceptibility of cancer cells with enhanced mitochondrial–ER tethering and, in parallel, their persistent need for mitochondrial-ER communication, causing an inverse correlation between ER-mitochondrial stability and UCP2 expression.

### Inverse Correlation Between UCP2 and Proteins Stabilizing Mitochondrial-ER Interaction as a Survival Strategy of Cancer Cells

**Figure 3:**
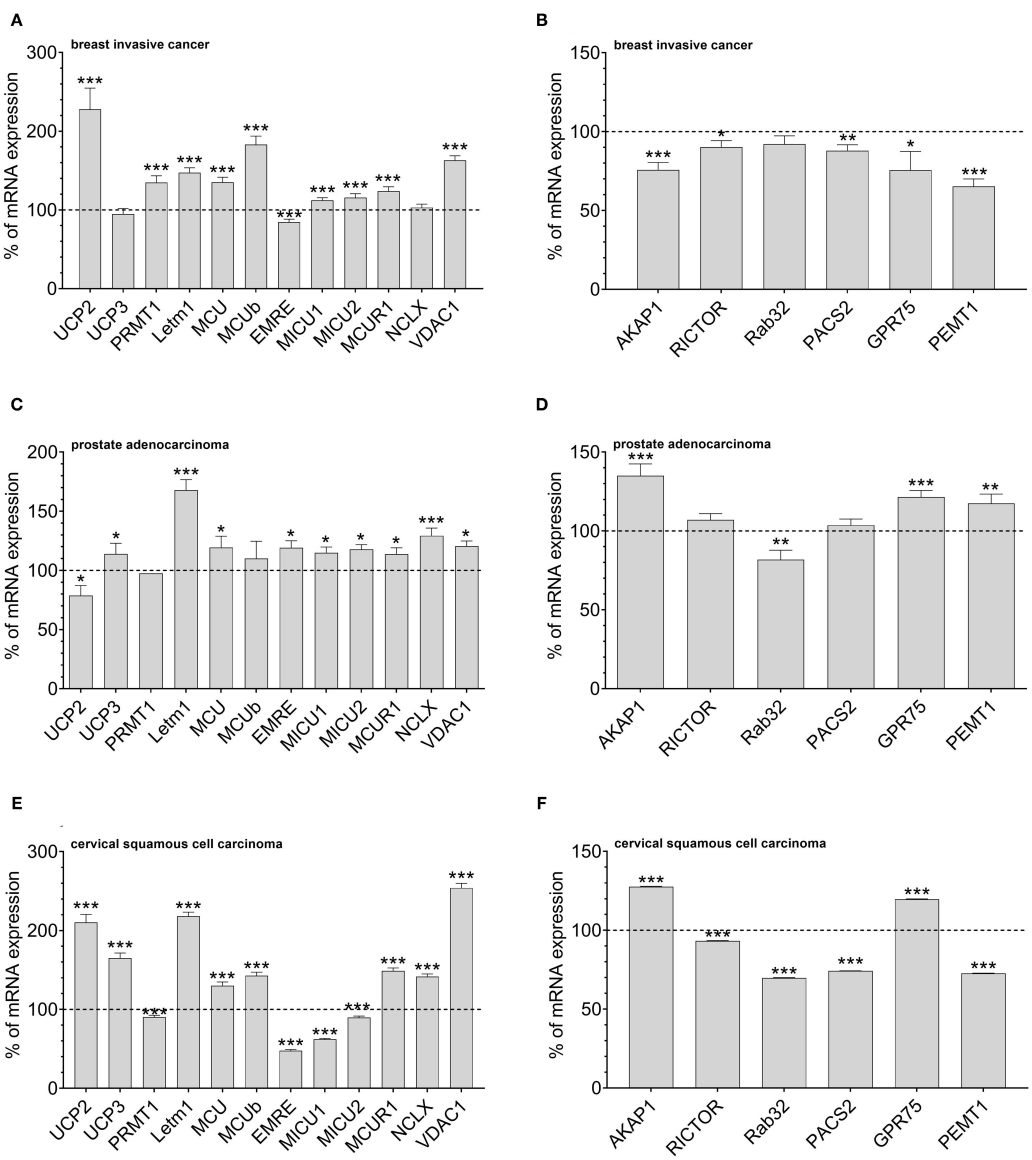
Mrna expression analysis of data obtained from TCGA. mRNA expression levels of proteins involved in mitochondrial Ca^2+^ uptake, including UCP2, UCP3, LETM1, MCU, MCUb, EMRE, MICU1, MICU2, MCUR1, and NCLX, mitochondrial Ca^2+^ extrusion such as NCLX in breast invasive cancer tissues, determined via TCGA analysis and presented as bar graphs, normalized to mRNA expression levels in adjacent normal tissue (*n* = 114) **(A)**. mRNA expression levels of proteins stabilizing contact sites between mitochondria and ER, including AKAP1, RICTOR, Rab32, PACS2, GPR75, and PEMT1, in breast invasive cancer tissues, determined via TCGA analysis and presented as bar graphs, normalized to mRNA expression levels in adjacent normal tissue (*n* = 114) **(B)**. mRNA expression levels of proteins involved in mitochondrial Ca^2+^ uptake, including UCP2, UCP3, LETM1, MCU, MCUb, EMRE, MICU1, MICU2, MCUR1, and NCLX, mitochondrial Ca^2+^ extrusion such as NCLX in prostate adenocarcinoma tissues, determined via TCGA analysis and presented as bar graphs, normalized to mRNA expression levels in adjacent normal tissue (*n* = 52) **(C)**. mRNA expression levels of proteins stabilizing contact sites between mitochondria and ER, including AKAP1, RICTOR, Rab32, PACS2, GPR75, and PEMT1, in prostate adenocarcinoma tissues, determined via TCGA analysis and presented as bar graphs, normalized to mRNA expression levels in adjacent normal tissue (*n* = 52) **(D)**. mRNA expression levels of proteins involved in mitochondrial Ca^2+^ uptake, including UCP2, UCP3, LETM1, MCU, MCUb, EMRE, MICU1, MICU2, MCUR1, and NCLX, mitochondrial Ca^2+^ extrusion such as NCLX in cervical squamous cell carcinoma tissues (*n* = 303), determined via TCGA analysis and presented as bar graphs, normalized to the mean of mRNA expression levels in adjacent normal tissue (*n* = 3) **(E)**. mRNA expression levels of proteins stabilizing contact sites between mitochondria and ER, including AKAP1, RICTOR, Rab32, PACS2, GPR75, and PEMT1, in cervical squamous cell carcinoma tissues (*n* = 303), determined via TCGA analysis and presented as bar graphs, normalized to the mean of mRNA expression levels in adjacent normal tissue (*n* = 3) **(F)**. Significant differences were assessed via unpaired *t-*test and presented as specific *p*-values (*p ≤ 0.05, **p ≤ 0.01, ***p ≤ 0.001).

According to our data that show an adaptation in UCP2 expression in cancer cells with fostered mitochondria–ER tethering, we assumed that cancer cell might balance mitochondrial Ca^2+^ loading and the organelles' interaction by regulation of expression levels of UCP2 and proteins stabilizing mitochondrial-ER interaction. Therefore, we investigated mRNA expression levels of proteins involved in mitochondrial Ca^2+^ uptake and the interaction between mitochondria and ER in tissues from cervical squamous cell carcinoma, breast invasive cancer and prostate adenocarcinoma by analyzing data from The Cancer Genome Atlas (TCGA). MCU, its dominant-negative form MCUb, LETM1, and VDAC, were found to be upregulated in breast invasive cancer. However, the most pronounced upregulation was found for UCP2 (Figure 3A). Notably, expression levels of proteins facilitating mitochondrial-ER interaction, including AKAP1, RICTOR, Rab32, PACS2, GPR75, and PEMT1, were reduced in these cancer tissues (Figure 3B). In contrast, in prostate adenocarcinoma tissues, UCP2 expression is reduced (Figure 3C), while several MAM-stabilizing proteins, including AKAP1, RICTOR, GPR75, and PEMT1, were found to be upregulated (Figure 3D). While expression levels in normal and tumor tissue samples of individual patients were matched for breast invasive cancer and prostate adenocarcinoma, just a limited number of expression levels was available for normal tissues of cervical squamous cell carcinoma patients. Consequently, we compared expression levels of cervical squamous cell carcinoma tissue samples with the mean expression level of available adjacent normal tissues. We found similar results as for breast invasive cancer, including an upregulation of UCP2 (Figure 3E) and a significant downregulation of four out of six genes associated with mitochondrial-ER interaction stabilization (Figure 3F). In summary, we found a tendency to an inverse correlation of UCP2 and proteins stabilizing mitochondrial-ER interaction in all three investigated cancer types.

### ER Stress-Induced Stability Increase in ER-Mitochondrial Interplay and Downregulation of UCP2

**Figure 4:**
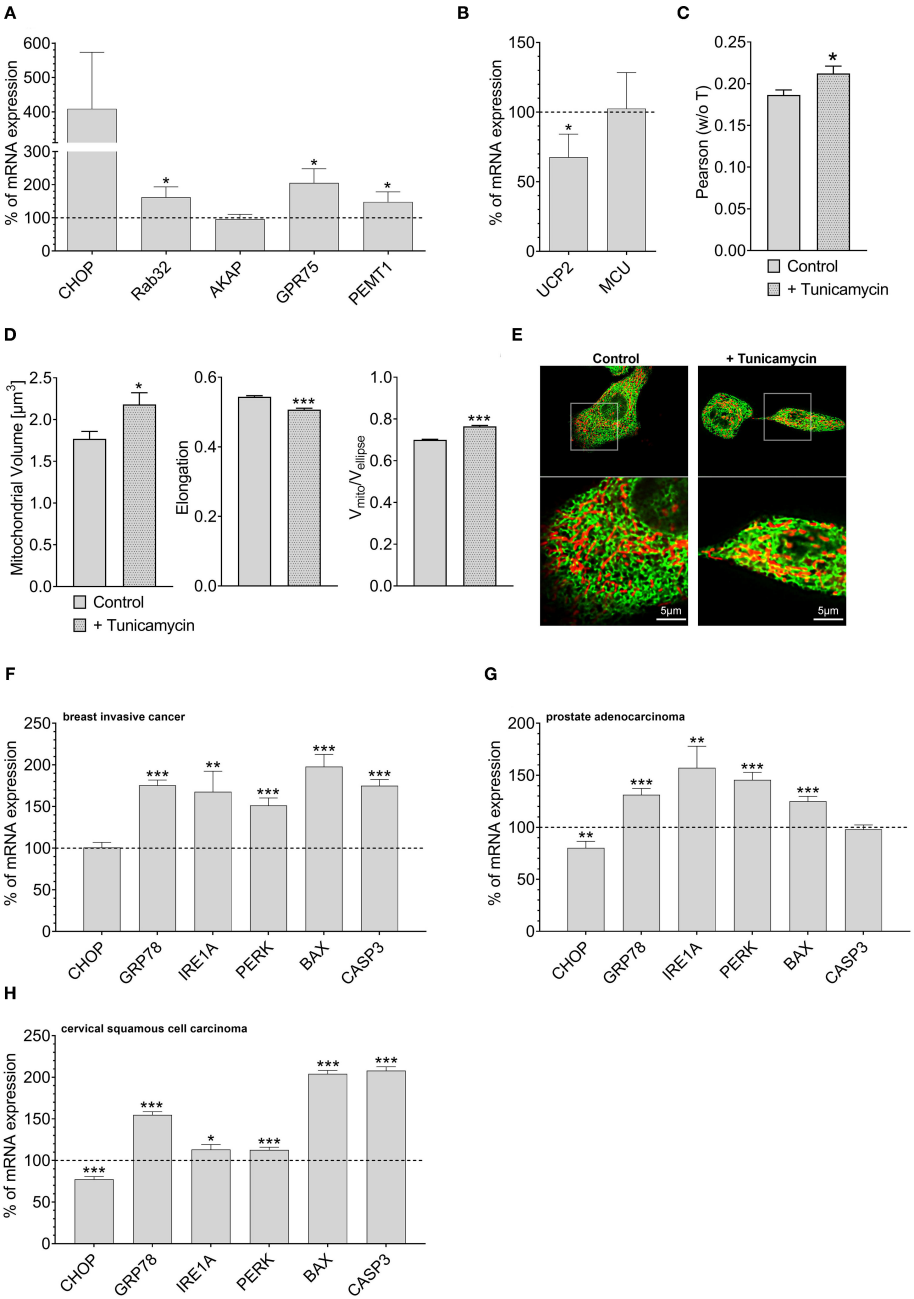
Mrna expression levels of ER stress markers. mRNA expression ratios of CHOP, Rab32, AKAP1, GPR75, PEMT1, GRP78, and MFN2 in HeLa cells after treatment with 0.6 μM tunicamycin for 4 h in comparison to untreated HeLa cells (*n* = 3) **(A)**. mRNA expression ratios of UCP2 and MCU in HeLa cells after treatment with 0.6 μM tunicamycin for 4 h compared to untreated HeLa cells (*n* = 3) **(B)**. Pearson coefficient showing colocalization of mitochondria and ER of untreated HeLa cells (*n* = 160/18) and HeLa cells treated with 0.6 μM tunicamycin for 12 h (*n* = 166/19) **(C)**. *Left* bar graph reflects mitochondrial volume [μm^3^], *middle* bar graph elongation and *right* bar graph V_mito_/V_ellipse_ of untreated HeLa cells (*n* = 160/18) and HeLa cells treated with 0.6 μM tunicamycin for 12 h (*n* = 166/19) **(D)**. Representative confocal images of untreated HeLa cells and HeLa cells treated with 0.6 μM tunicamycin for 12 h expressing D1ER (green) and stained with MitoTrackerRed® CMXRos (red) **(E)**. mRNA expression levels of proteins involved in ER stress, including CHOP, GRP78, IRE1A, PERK, BAX, and CASP3, in breast invasive cancer tissues, determined via TCGA analysis and presented as bar graphs, normalized to mRNA expression levels in adjacent normal tissue (*n* = 114) **(F)**. mRNA expression levels of proteins involved in ER stress, including CHOP, GRP78, IRE1A, PERK, BAX, and CASP3, in prostate adenocarcinoma tissues, determined via TCGA analysis and presented as bar graphs, normalized to mRNA expression levels in adjacent normal tissue (*n* = 52) **(G)**. mRNA expression levels of proteins involved in ER stress, including CHOP, GRP78, IRE1A, PERK, BAX, and CASP3, in cervical squamous cell carcinoma tissues (*n* = 303), determined via TCGA analysis and presented as bar graphs, normalized to the mean of mRNA expression levels in adjacent normal tissue (*n* = 3) **(H)**. Significant differences were assessed via unpaired *t-*test and presented as specific *p*-values (*p ≤ 0.05, **p ≤ 0.01, ***p ≤ 0.001).

Overexpression of Rab32 might be considered as a highly artificial manipulation of cellular homeostasis. Therefore, we investigated whether a physiological process might also trigger an inverse correlation between UCP2 and proteins involved in mitochondrial-ER interaction. Induction of ER stress by 0.6 μM tunicamycin treatment for 4 h has already been reported to induce enhanced mitochondrial-ER interaction (Madreiter-Sokolowski et al., 2019b). Indeed, tunicamycin treatment caused an upregulation of MAM-stabilizing proteins such as Rab32, GPR75, and PEMT1 (Figure 4A), and reduced the expression of UCP2. The expression of MCU did not change significantly (Figure 4B). Respective colocalization analysis confirmed increased contact between mitochondria and ER in HeLa cells treated with tunicamycin overnight (Figures 4C,E). ER stress resulted in increased mitochondrial volume, less elongation and reduced branching (Figure 4D). Notably, several ER stress markers, including GRP78, IRE1A, PERK, BAX, and CASP3 were also found to be upregulated in breast invasive cancer (Figure 4F), prostate adenocarcinoma (Figure 4G) and cervical squamous cell carcinoma (Figure 4H), a potential hint that ER stress in cancer cells might induce alterations in the mitochondrial-ER interplay and the expression of UCP2 in cancer.

## Methods

### Cell Culture, Transfection and Treatment

Cancer cell lines were grown in Dulbecco's Modified Eagle Medium (DMEM) from Sigma Aldrich (Vienna, Austria). Media were supplemented with 10% fetal bovine serum, 100 U/ml penicillin, 100 μg/ml streptomycin, and 1.25 μg/ml amphotericin B (Gibco, Lifetechnologies; Vienna, Austria). Cells were transiently transfected at a confluence of 60–80% with 1.5 μg appropriate plasmid DNA and 100 μM of respective siRNA using 2.5 μl of TransFast™ transfection reagent (Promega; Madison, WI, US) in 1 ml of serum- and antibiotic-free medium. The UCP2 plasmid used for overexpression, the respective siRNA against UCP2 as well as the transfection procedure applied have already been tested in regard to their transfection efficacy (Trenker et al., 2007). The morphological changes in ER-mitochondrial colocalization induced by the respective overexpression of AKAP-CAAX-RFP and Rab32 were verified by confocal microscopy (Figure 1). The transfection mix was replaced by full culture medium after 24 h. All experiments were performed 48 h after transfection. BAPTA-AM [2 μM] and EGTA-AM [2 μM] treatment was done 45 min prior live-cell imaging experiments.

### mRNA Isolation and qRT-PCR

Total RNA from cells was isolated using the PEQLAB total RNA isolation kit (Erlangen, Germany), and reverse transcription was performed using a cDNA synthesis kit (Applied Biosystems; Foster City, CA). qRT-PCR was performed using QuantiFast SYBR Green RT-PCR kit (Qiagen; Hilden, Germany) as described previously (Madreiter-Sokolowski et al., 2016a). Primers for qRT-PCR were obtained from Invitrogen (Vienna, Austria).

### Cell Viability and Apoptosis Assay

For cell viability and apoptosis assays, cells were plated 24 h after transfection on 96-well-plates at a density of 5,000 cells/well. Cell viability was measured 48 h after transfection using CellTiter-Blue assay and apoptotic caspase activity using Caspase-Glo® 3/7 assay (Promega; Madison, WI, US) as reported previously (Madreiter-Sokolowski et al., 2016b).

### Live-Cell Imaging Experiments

Dynamic changes in [Ca^2+^]_mito_ were followed in cells expressing 4mtD3cpv. Medium was removed and cells were kept in loading buffer containing 135 mM NaCl, 5 mM KCl, 2 mM CaCl_2_, 1 mM MgCl_2_, 10 mM Hepes, 2.6 mM NaHCO_3_, 440 μM KH_2_PO_4_, 340 μM Na_2_HPO_4_, 10 mM D-glucose, 0.1% vitamins, 0.2% essential amino acids, 1% penicillin/streptomycin, pH adjusted to 7.4. Single cell measurements were performed under constant perfusion (PS9, NGFI, Graz, Austria) using a perfusion chamber with mounted 3 cm (Ø) glass-coverslip (PC30, NGFI) on a Zeiss AxioVert inverted microscope (Zeiss; Göttingen, Germany) equipped with a polychromator illumination system (VisiChrome, Visitron Systems; Puchheim, Germany) and a thermoelectric-cooled CCD camera (Photometrics CoolSNAP HQ, Visitron Systems; Puchheim, Germany). Transfected cells were imaged with a 40 × oil-immersion objective (Zeiss). Excitation of the FRET-based genetically encoded Ca^2+^ indicator 4mtD3cpv was at 440 ± 10 nm (440AF21, Omega Optical; Brattleboro, VT, USA), and emissions were recorded at 480 and 535 nm using emission filters (480AF30 and 535AF26, Omega Optical) mounted on a filterwheel (Ludl Electronic Products, Hawthorne, NY, US). Devices were controlled and data were acquired by VisiView 2.0.3 (Visitron Systems) software and analyzed with GraphPad Prism version 5.00 for Windows (GraphPad Software; San Diego, CA). Results of FRET measurements are shown as (Ri—Background) + [(Ri—Background)—(R0—Background)], where R0 is the basal ratio, to correct for photobleaching and/or photochromism.

### Co-localization Analysis

D1ER-overexpressing HeLa cells were stained for 10 min with 200 nM MitoTracker® Red CMXRos and imaged directly. High-resolution images of cells were recorded by using a confocal spinning disk microscope (Axio Observer.Z1 from Zeiss, Gottingen, Germany) equipped with 100x objective lens (Plan-Fluor x100/1.45 Oil, Zeiss), a motorized filter wheel (CSUX1FW, Yokogawa Electric Corporation, Tokyo, Japan) on the emission side, AOTF-based laser merge module for laser line 405, 445, 473, 488, 561, and 561 nm (Visitron Systems) and a Nipkow-based confocal scanning unit (CSU-X1, Yokogawa Electric corporation). The D1ER and Mitotracker® Red CMXRos were alternately excited with 488 and 561 nm laser lines, respectively, and emissions were acquired at 353 and 600 nm using a charged CCD camera (CoolSNAP-HQ, Photometrics, Tucson, AZ, USA). Z-stacks of both channels in 0.2 μm increments were recorded. The software VisiView acquisition software (Universal Imaging, Visitron Systems) was used to acquire the imaging data. Images were blind deconvoluted with NIS-elements (Nikon, Austria). The colocalization was determined on a single cell level using ImageJ and the plugin coloc2. The Pearson coefficient and the Costes thresholded Manders coefficient were calculated.

### 3D Morphological Analysis of Mitochondria

Image stacks were deconvoluted through blind deconvolution (NIS-Elements, Nikon, Austria). Morphology parameters were measured automatically via a custom-made ImageJ macro using the following procedure. An additional background subtraction based on the rolling ball method was introduced to further enhance contrast for later analysis. Both, a global auto Otsu threshold using a stack histogram as well as a local auto Otsu threshold (radium of 640 nm) based on a single slice histogram, were applied to the stack and merged. To exclude not transfected cells, D1ER staining was thresholded and used as a mask for MitoTracker® Red CMXROS stained mitochondria. Binarized mitochondria got segmented via the ImageJ plugin 3D manager. Mitochondrial volume and surface were determined through the plugin 3D Geometrical Measure. The Plugin 3D Ellipsoid Fitting generated an ellipsoid fit of mitochondria to measure elongation and flatness parameters. Further, the mitochondrial branching was determined by dividing the volume of each mitochondrion by the volume of each respective fitted ellipsoid (V_mito_/V_ellipse_). The bigger the ratio, the smaller the rate of mitochondrial branching in the present sample.

### Data Acquisition via Xena

The mRNA expression levels of the respective genes in various tumor tissues were obtained as RNA SeqV2 RSEM values through UCSC Xena (https://xenabrowser.net/) as The Cancer Genome Atlas (Provisional, TCGA) datasets in September 2020. The selected genomic profile was “gene expression RNAseq (polyA+ IlluminaHiSeq),” and the entered gene set was a user-defined list. Expression levels of normal and tumor tissue samples with the same sample ID were matched (Kammerer et al., 2016). The mRNA expression levels of breast invasive cancer and prostate adenocarcinoma tissues were normalized to the mRNA expression levels of corresponding adjacent healthy tissue samples from the same patient (100% value). Patients with missing expression levels of normal or tumor tissues were excluded. The mRNA expression levels of cervical squamous cell carcinoma were normalized to the mean mRNA expression levels of available adjacent healthy tissue samples.

### Statistical Analysis

The statistical analysis was performed with GraphPad Prism 5.0 using one-way ANOVA or unpaired Student's *t*-test, if applicable, and *p* < 0.05 was considered to be significant.

## Discussion

This paper was designed to investigate how cancer cells fine-tuning and coordinate mitochondrial-ER tethering and mitochondrial Ca^2+^ uptake to balance mitochondrial Ca^2+^ levels. Mitochondrial Ca^2+^ is essential for stimulation of mitochondrial metabolism (Mammucari et al., 2018), while mitochondrial Ca^2+^ overload leads to mitochondrial dysfunction and, ultimately, cell death (Raffaello et al., 2016; Madreiter-Sokolowski et al., 2018). Consequently, several studies revealed an essential role of mitochondrial Ca^2+^ in cancer cell migration (Tang et al., 2015), invasiveness (Tosatto et al., 2016), and cancer growth (Liu et al., 2020), while others reported cell death induced by mitochondrial Ca^2+^ accumulation (Yoon et al., 2014; Madreiter-Sokolowski et al., 2016b).

Regions of ER-mitochondrial contact sites are signaling hubs for cellular survival, metabolism, and tumorigenesis, housing numerous cancer-related proteins, including oncogenes and tumor suppressors (Doghman-Bouguerra and Lalli, 2019). Since constitutive Ca^2+^ flux from the ER to mitochondria crucially affects cancer cells (Cardenas et al., 2016; Madreiter-Sokolowski et al., 2016b), we manipulated ER-mitochondrial interaction to investigate its impact on mitochondrial Ca^2+^ uptake. In the current study, we used the AKAP-RFP-CAAX construct to disrupt (Madreiter-Sokolowski et al., 2016b) and Rab32 to foster (Bui et al., 2010) mitochondria-ER tethering. Our findings that mitochondrial Ca^2+^ uptake in cells with reduced mitochondrial–ER contacts was not affected by the modulation of UCP2 expression point to the distinct involvement of UCP2 in mitochondrial Ca^2+^ uptake exclusively in regions with MAMs. This assumption is further supported by data showing that the impact of UCP2 knockdown on mitochondrial Ca^2+^ sequestration is increased in cells with enforced mitochondria-ER tethering. These data are in line with previous reports that described UCP2 as being exclusively engaged in MAM-located mitochondrial Ca^2+^ uptake (Waldeck-Weiermair et al., 2010, 2011). Notably, the contribution of UCP2 to mitochondrial Ca^2+^ uptake (Trenker et al., 2007) is limited to conditions of enhanced PRMT1 activity and subsequent methylation of MICU1, as the case in most cancer cells (Madreiter-Sokolowski et al., 2016a).

Interestingly, HeLa cells that survived artificial enforcement of mitochondria–ER tethering significantly reduced mRNA expression levels of UCP2. Accordingly, we speculate that an inverse correlation between the expression of UCP2 and proteins stabilizing the mitochondrial-ER contact exists, reflecting an adaptation strategy of cancer cells to prevent mitochondrial Ca^2+^ overload. The mRNA expression pattern of breast invasive cancer and prostate adenocarcinoma tissues further strengthened this hypothesis. An upregulation of UCP2 has already been reported for several cancer types, including breast invasive cancer, thyroid carcinoma, lung carcinoma, bladder urothelial cancer, and colorectal adenocarcinoma (Madreiter-Sokolowski et al., 2017b). Hence, UCP2' impact on the MCUC in case of elevated PRMT1 activity is further demonstrated by the poor survival prognosis of lung carcinoma patients with combined upregulation of UCP2 and PRMT1 (Madreiter-Sokolowski et al., 2017b). Besides PRMT1-mediated methylation of MICU1, several posttranslational modifications are reported for proteins involved in the MCUC, including modification of MCU by Ca^2+^/calmodulin-dependent protein kinase II (CamKII) (Joiner et al., 2012), protein tyrosine kinase Pyk2 (O-Uchi et al., 2014), ROS (Dong et al., 2017), and metabolites (Nemani et al., 2020; Tomar and Elrod, 2020). Moreover, phosphorylation of NCLX by protein kinase A (PKA) was found to reverse mitochondrial Ca^2+^ overload (Kostic et al., 2015).

The process of fine-tuning mitochondrial Ca^2+^ uptake by PRMT1-driven methylation of MICU1 is assumed to take several minutes (Qian et al., 2018). In contrast, the adaptation of mitochondrial Ca^2+^ homeostasis by UCP2 protein expression is most probably delayed for several hours (Troscher et al., 2019). Therefore, we assume that enhanced expression of UCP2, which is found in most human cancer types (Madreiter-Sokolowski et al., 2017b), long lastingly facilitates mitochondrial ATP production by Ca^2+^-mediated stimulation of Krebs cycle dehydrogenases. Notably, a balanced modulation of MCUC activity by downregulation of MCU, induced by overexpression of cancer-related miR-25, has been also found in colon cancer (Marchi et al., 2013). Moreover, the mitochondrial tumor suppressor Fus1 diminishes MCU expression (Uzhachenko et al., 2014) and the expression of NCLX affects colorectal cancer growth and metastasis (Pathak et al., 2020). A similar transcriptional regulation of proteins involved in the MCUC was found in neurons as a neuroprotective strategy against mitochondrial Ca^2+^ overload-induced neuronal cell death (Qiu et al., 2013; Muhling et al., 2014). In addition, increased expression of the gatekeeper MICU1, induced by the transcription factor early growth response 1 (EGR1), was reported to protect against mitochondrial Ca^2+^ overload (Nemani et al., 2020).

Altogether it is tempting to speculate that the machinery of proteins achieving mitochondrial Ca^2+^ homeostasis is subject to intense translational and posttranslational modifications, reflecting the fundamental importance of balancing mitochondrial Ca^2+^ homeostasis under conditions of various metabolism states, and diseases. In line with this hypothesis, fusion and fission of mitochondria and dynamical changes of the ER occur within few seconds (Liu et al., 2009; Costantini and Snapp, 2013). Modulation of ER-mitochondrial tethering might be a powerful and necessary tool to rapidly adjust the amount of Ca^2+^ taken up by mitochondria. Consequently, we speculate that the contact intensity between mitochondria and ER is under dynamical change in cancer cells, continuously adjusting mitochondrial Ca^2+^ uptake to the cell's energy demands. In this regard, the dynamin-related protein DRP1, involved in Ca^2+^-dependent mitochondrial fission and localized to mitochondrial-ER contact sides (Cereghetti et al., 2008), might play a role in dynamic short-term mitochondrial Ca^2+^ homeostasis by capping ER-mitochondrial interaction after ER Ca^2+^ release (Friedman et al., 2011). On the other hand, UCP2 knockdown results in more stable and elongated mitochondria, probably associated with a lower rate of mitochondrial fission and enhanced tethering with the ER (Hass and Barnstable, 2019). In summary, these reports might point to an UCP2-dependent regulation of mitochondrial Ca^2+^ homeostasis by modulation of mitochondrial fission.

It has been previously shown that the enforcement of mitochondria–ER interplay is often associated with enhanced ER stress (Madreiter-Sokolowski et al., 2019b). We found ER stress markers upregulated in breast invasive cancer tissues with high expression of UCP2 and prostate adenocarcinoma with low expression of UCP2. Accordingly, we speculate that conditions of ER stress, often associated with an uncontrolled ER Ca^2+^ leak (Oakes et al., 2005), might trigger an inverse correlation between UCP2 and proteins stabilizing ER-mitochondrial interactions.

According to our data presented herein, targeting a well-balanced mitochondrial Ca^2+^ homeostasis by disturbance of proper mitochondrial Ca^2+^ uptake or by affecting the intensity of the mitochondrial-ER interplay might be a promising strategy to kill cancer cells specifically. Moreover, modulation of Ca^2+^ signaling was found to increase the responsiveness of cancer cells toward chemotherapeutics (Kerkhofs et al., 2018). Consequently, targeting mitochondrial Ca^2+^ signaling might serve as option to re-sensitize cancer cells, which escaped the cytotoxic effect of chemotherapeutics. Further studies using various cancer cell lines as well as respective tumor tissues, including also the most common cancer types like breast, prostate and lung cancer, are urgently needed to gain a better understanding about the interplay between proteins stabilizing ER-mitochondrial linkage and proteins involved in mitochondrial Ca^2+^ uptake and its impact on cancer cell metabolism, survival, proliferation and on susceptibility to chemotherapeutics.

## Data Availability Statement

The raw data supporting the conclusions of this article will be made available by the authors, without undue reservation.

## Author Contributions

CM-S and WG designed this study. CM-S performed live cell imaging experiments, qRT-PCR analysis, cell viability, and apoptosis assays. BG analyzed the colocalization between mitochondria and ER. AS performed clinical data analysis. CM-S, RM, and WG wrote the manuscript. All authors contributed to the article and approved the submitted version.

## Conflict of Interest

The authors declare that the research was conducted in the absence of any commercial or financial relationships that could be construed as a potential conflict of interest.
